# Novel Angiographic Scores for evaluation of Large Vessel Vasculitis

**DOI:** 10.1038/s41598-018-34395-7

**Published:** 2018-10-29

**Authors:** Enrico Tombetti, Claudia Godi, Alessandro Ambrosi, Frances Doyle, Alana Jacobs, Allan P. Kiprianos, Taryn Youngstein, Katie Bechman, Angelo A. Manfredi, Ben Ariff, Justin C. Mason

**Affiliations:** 1 Vascular Sciences/Rheumatology, Imperial College London and Imperial College Healthcare NHS Trust, Hammersmith Hospital, Du Cane Rd, W12 0HS London, UK; 2 Università Vita-Salute San Raffaele, Via Olgettina 58, 20132 Milan, Italy; 3 Department of Biomedical and Clinical Sciences “L. Sacco”, Milan University, Milan, Italy; 4 IRCCS Ospedale San Raffaele, Via Olgettina 60, 20132 Milan, Italy; 5 Imaging Department, Imperial College Healthcare NHS Trust, Du Cane Rd, W12 0HS London, UK

## Abstract

Arterial involvement is the cardinal feature of large-vessel vasculitis (LVV) and prevention of disease progression is the principal therapeutic goal. However, development of tools for its evaluation represents a major unmet need. To address this, a widely-applicable imaging tool for LVV, analysing arterial involvement in 17 arterial territories, has been developed and validated. Individual stenosis and dilation scores were generated and combined in a composite score. The methodology was validated cross-sectionally and longitudinally in 131 patients, 96 Takayasu arteritis (TA), 35 large-vessel giant-cell arteritis (LV-GCA). In total, 4420 arterial segments from 260 imaging studies were evaluated. The new scores allowed quantitative grading of LVV arterial involvement with high consistency, revealing inter-patient differences. TA had higher stenosis and composite scores and lower dilation scores than LV-GCA. Baseline stenotic and composite scores reflected arterial damage rather than disease-activity. Longitudinal changes in all three scores correlated with disease activity and mirrored arterial disease evolution, reflecting both progressive injury and lesion improvement. Increases ≥1 in any score were specific for arterial disease progression. The scores objectively quantify arterial involvement in LVV, providing precise definition of disease phenotype and evolution. We propose that they represent novel vascular outcome measures essential for future clinical trials.

## Introduction

Large-vessel vasculitides (LVVs) are characterised by idiopathic arterial inflammation predisposing to vascular remodelling with stenosis/occlusion or dilation^1–3^. The two predominant LVVs are Takayasu arteritis (TA) and giant-cell arteritis (GCA), typically affecting young and elderly patients, respectively^2^. When compared to the progress made in recent decades in other inflammatory rheumatic diseases, clinical advances in LVV have been incremental and management remains sub-optimal^4^. Although biologic therapies for GCA^5^ and TA^6,7^ are beginning to change the perspective, their use raises important issues. These include the limitations associated with current disease activity assessments based on clinical symptoms or acute-phase reactants (C-reactive protein (CRP) and erythrocyte-sedimentation rate (ESR))^8–12^ and in the multi-item measures including the National Institute of Health (NIH) criteria and Indian Takayasu Activity Score (ITAS)^11,13^. Symptom suppression does not necessarily reflect remission of arterial wall inflammation and silent arterial disease progression has been reported, especially during tocilizumab therapy^9,14,15^. Thus, imaging is required for diagnosis and serially every 6–12 months to monitor arterial remodeling in those with persistently active or grumbling disease. However, imaging is not routinely recommended for patients in clinical and biochemical remission with stable imaging studies^12,16,17^.

Arterial injury directly impacts LVV clinical features and prognosis^4^. Prevention of arterial disease progression is the fundamental therapeutic goal^12^. However, evidence demonstrating the effects of immunosuppressive therapy remains relatively sparse. Clinical trial outcomes have predominantly reflected changes in disease activity, with the imaging assessment of arterial disease incorporated as a secondary end-point^5–7^. This omission reflects the lack of standardised and validated non-invasive imaging outcome measures^18^. The recent report from the OMERACT group stresses the urgent need for defined outcome measures for LVV^19^. Likewise, the 2018 EULAR LVV Imaging Guidelines specifically recommend the development of ‘standardised, well-validated scoring methodologies for all imaging modalities’ and ‘composite scores for imaging-based monitoring of patients with LVV’^17^.

These well-recognised challenges formed the basis of the current study. A novel imaging-based scoring algorithm has been designed to allow precise qualitative and quantitative definition of arterial involvement in LVV based on luminal changes. Additional goals were wide applicability and utility for both cross-sectional and longitudinal disease assessment in future clinical trials. To achieve this, validated methods for assessing carotid artery atherosclerosis were modified to account for specific LVV features. Three new scores were generated: a Stenosis and a Dilation Score, which were combined in a Composite Score. The three scores were then tested in a large LVV cohort to provide cross-sectional and longitudinal validation.

## Results

### Development of arterial injury scores

**Figure 1:**
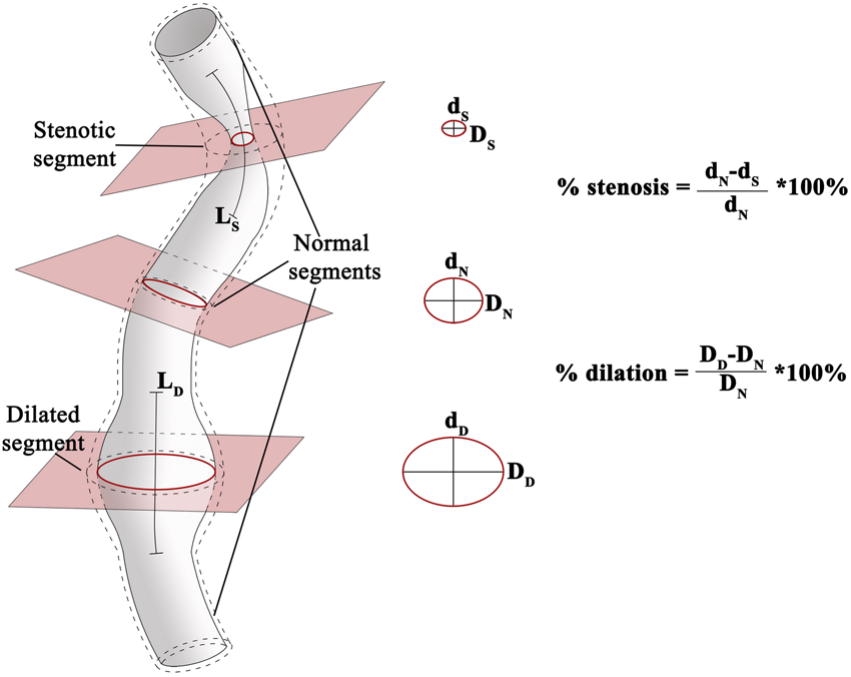
Multi-planar navigation to score arterial stenosis and dilation. A model artery showing stenosis and dilatation with interposed normal segments. Percent stenosis and dilation, and the length of stenotic (L_S_) and dilated (L_D_) segments are assessed with multi-oblique navigation: the planes of maximum stenosis and dilation are identified, together with a plane with a normal lumen. A valid reference plane excludes significant branching between the index and the reference planes. Orthogonality to the arterial axis is guaranteed by multi-planar navigation. The central panels show the identification of the smallest and largest arterial diameters in the plane of maximum stenosis (d_S_ and D_S_), of maximal dilation (d_D_ and D_D_) and in the plane with a normal arterial lumen (d_N_ and D_N_).

The novel LVV scores were designed to be applicable to volumetric computed tomography-angiography (CTA) and magnetic resonance-angiography (MRA) studies. The scoring algorithm was derived from the well-validated North American Symptomatic Carotid Endarterectomy Trial (NASCET) and European Carotid Surgery Trial (ECST) methods. These scores utilise luminal data to define the severity of carotid atherosclerosis on the basis of percent stenosis^20,21^, but differ in the reference used to calculate percent stenosis (Supplementary Fig. 1). The detail behind the modification of NASCET and ECST for the assessment of LVV is included in the Methods section of the manuscript and is illustrated in Fig. 1. The modifications allowed assessment of a core-set of 17 arteries, selected on the basis of: (a) frequent involvement in LVV^10,22^ and (b) suitability for analysis by routine contrast-enhanced MRA and CTA (Table 1).

**Table 1:** Proposed scoring algorithm.

Stenosis - Scoring Individual Arteries			
Percent stenosis	Points	Length*	Extra-points
<50%	1	<1 cm	0
50–70%	2	1–3 cm	1
≥70%	3	3–10 cm	2
Functional occlusion	4	≥10 cm	3
**Dilation - Scoring Individual Arteries**			
**Percent dilation**	**Points**	**Length**	**Extra-points**
<50%	1	<1 cm	0
50–100%	2	1–3 cm	1
≥100% or needing	3	3-10 cm	2
Procedures		≥10 cm	3
**Core set of arteries**			
Ascending aorta		Left axillary artery	
Aortic arch		Left common carotid artery	
Thoracic aorta		Coeliac artery	
Abdominal aorta		Superior mesenteric artery	
Brachiocephalic artery		Right renal artery	
Right subclavian artery		Left renal artery	
Right axillary artery		Right common iliac artery	
Right common carotid artery		Left common iliac artery	
Left subclavian artery			
**Arterial Stenosis Score (ASS):** ∑ individual stenosis scores **Arterial Dilation Score (ADS):** ∑ individual dilation scores **Arterial Composite Score :** ASS+ADS			

*Lesion length was graded using a four-point scale: 0 for focal lesions (length < 10 mm), 1 for lesions above 10 and up to 30 mm, 2 for above 30 and up to 100 mm and 3 for >100 mm.

### Application of the scoring algorithm

**Figure 2:**
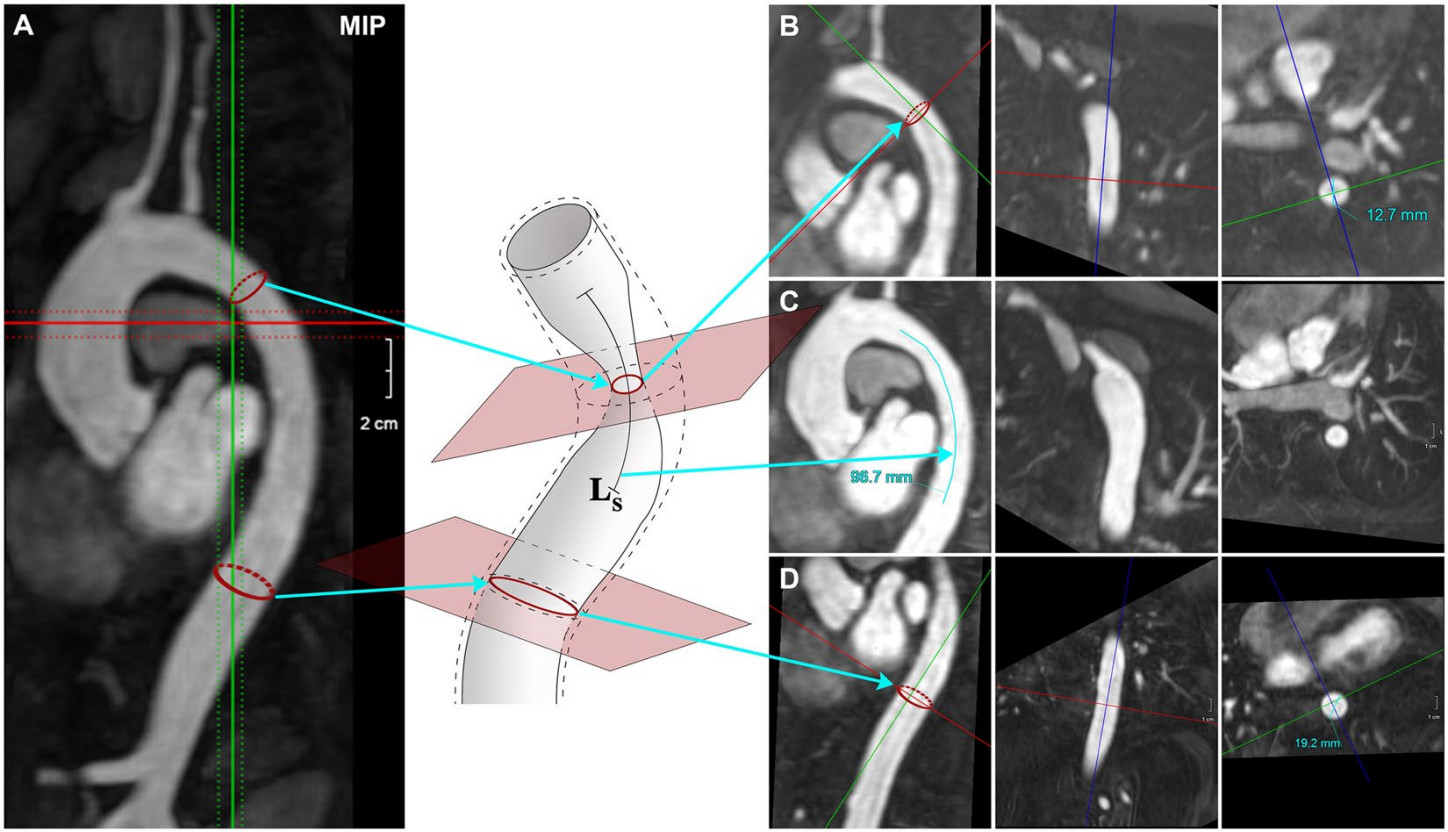
Scoring arterial involvement. (**A**) A stenotic lesion in the descending aorta in a maximum intensity projection (MIP) image using multi-oblique reconstructions. Coloured dotted lines show slice thickness and red ovals represent the artery at the planes of reference and maximum stenosis. (**B**) Measure of the smallest arterial diameter in the plane of maximum stenosis. (**C**) Assessment of lesion length. (**D**) Measure of the smallest diameter in a plane with a normal-appearing arterial lumen. Stenosis score of the descending aorta is 1 (<50% stenosis) +2 (length 30–100 mm) = 3; dilation score is 0.

**Figure 3:**
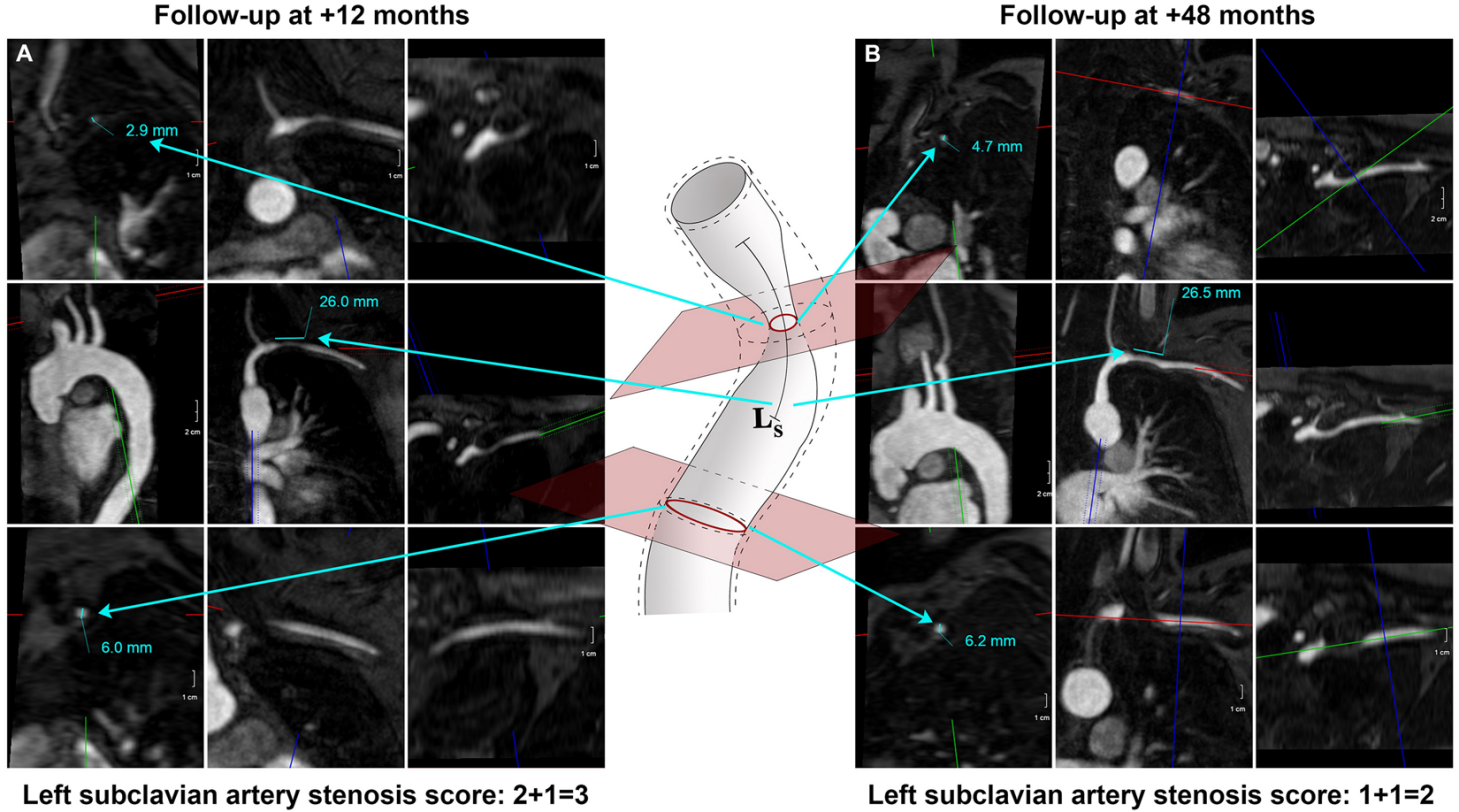
Longitudinal scoring of a left subclavian artery stenosis. The lesion presented is scored 12 and 48 months from baseline. (**A**) At 12 months, the percent stenosis was 50–70% and length 10–30 mm. The stenosis score was 2 + 1 = 3. (**B**) At 48 months, the percent stenosis is <50%, lesion length remains unchanged, and the stenosis score was 1 + 1 = 2.

The novel scoring algorithm was initially applied to the imaging studies of 131 consecutive LVV patients fulfilling enrolment criteria (96 TA, 35 LV-GCA), followed for 3.1 years (IQR 1.4–4.9). Two physicians jointly evaluated 260 scans and 4420 arterial segments, requiring on average 30–40 minutes per scan. Supplementary Table 1 shows descriptive statistics at baseline. Compared to LV-GCA, TA patients had longer disease duration, more extensive arterial damage and fewer were receiving corticosteroids. The stenotic, dilation and composite scores were generated in all cases. Illustrative examples are shown in Figs 2 and 3. A specific, independent subset analysis demonstrated a high level of scoring consistency (intra- and inter-observer reliability of the stenosis, dilation and composite scores of 0.999–0.996-0.996 and 0.995–0.993–0.992, respectively). Baseline scans (114 MRAs, 17 CTAs) showed a wide range of stenotic, dilation and composite scores (0–50, 0–26 and 0–53, respectively). These data demonstrate the full range of disease severity present in the cohort. Furthermore, they suggest that the scores have the potential to stratify individual patients based on the extent of arterial stenosis and dilation.

### The novel scores reflect disease phenotype heterogeneity

**Figure 4:**
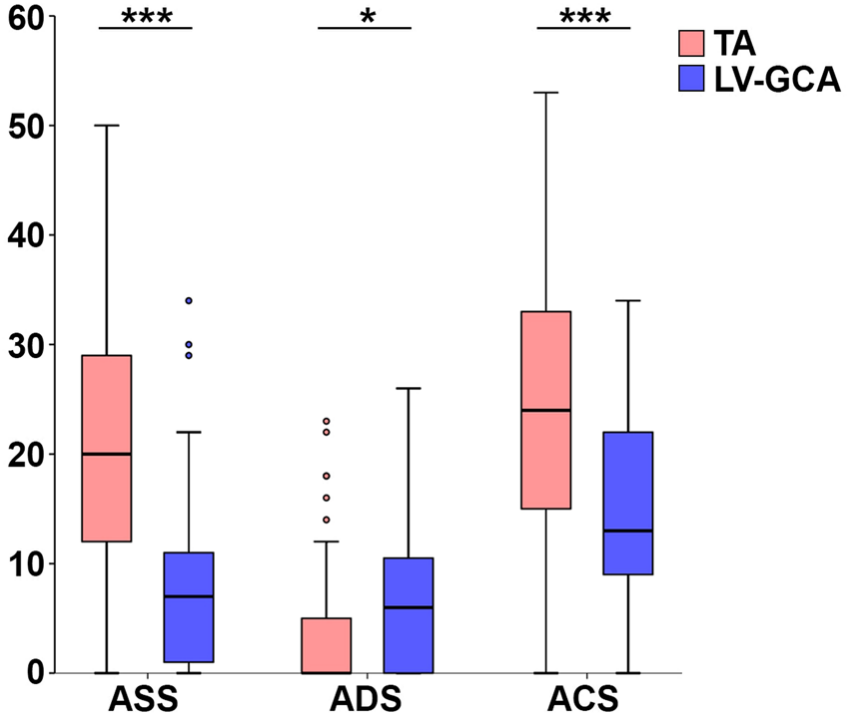
Differences between stenotic, dilation and composite score values in TA and LV-GCA. In comparison to LV-GCA, TA patients had higher stenosis (ASS) and composite scores (ACS) but lower dilation scores (ADS), (*p = 0.013, ***p < 0.001).

Next, the ability of the novel scores to distinguish disease heterogeneity was assessed. Compared to LV-GCA, TA patients had higher stenotic (median 20, IQR 12–29 Vs 7, IQR 1–11, p < 0.001) and composite scores (median 24, IQR 15–33 Vs 13, IQR 9–22, p < 0.001), but lower dilation scores (median 0, IQR 0–5 Vs 6, IQR 0–11, p = 0.013, Supplementary Table 1 and Fig. 4). Moreover, the scores reflected important features of disease phenotype. For example, 67 patients (51%) had a dilation score of 0, while four patients scored 0 for both stenotic and dilation scores. A qualitative assessment of the images derived from large arteries up to the carotid bifurcation confirmed that a dilation score of 0 accurately identified the patients without evidence of arterial dilation. A stenotic and dilation score of 0 was seen in the early “pre-angiographic” phase of LVV, diagnosed on the basis of a systemic inflammatory response and positive ^18^F-fluorodeoxyglucose PET imaging. These data show that the stenotic, dilation and composite scores can distinguish early and late phases of LVV and provide qualitative and quantitative definition of the arterial phenotype.

### Cross-sectional validation against damage indices

**Figure 5:**
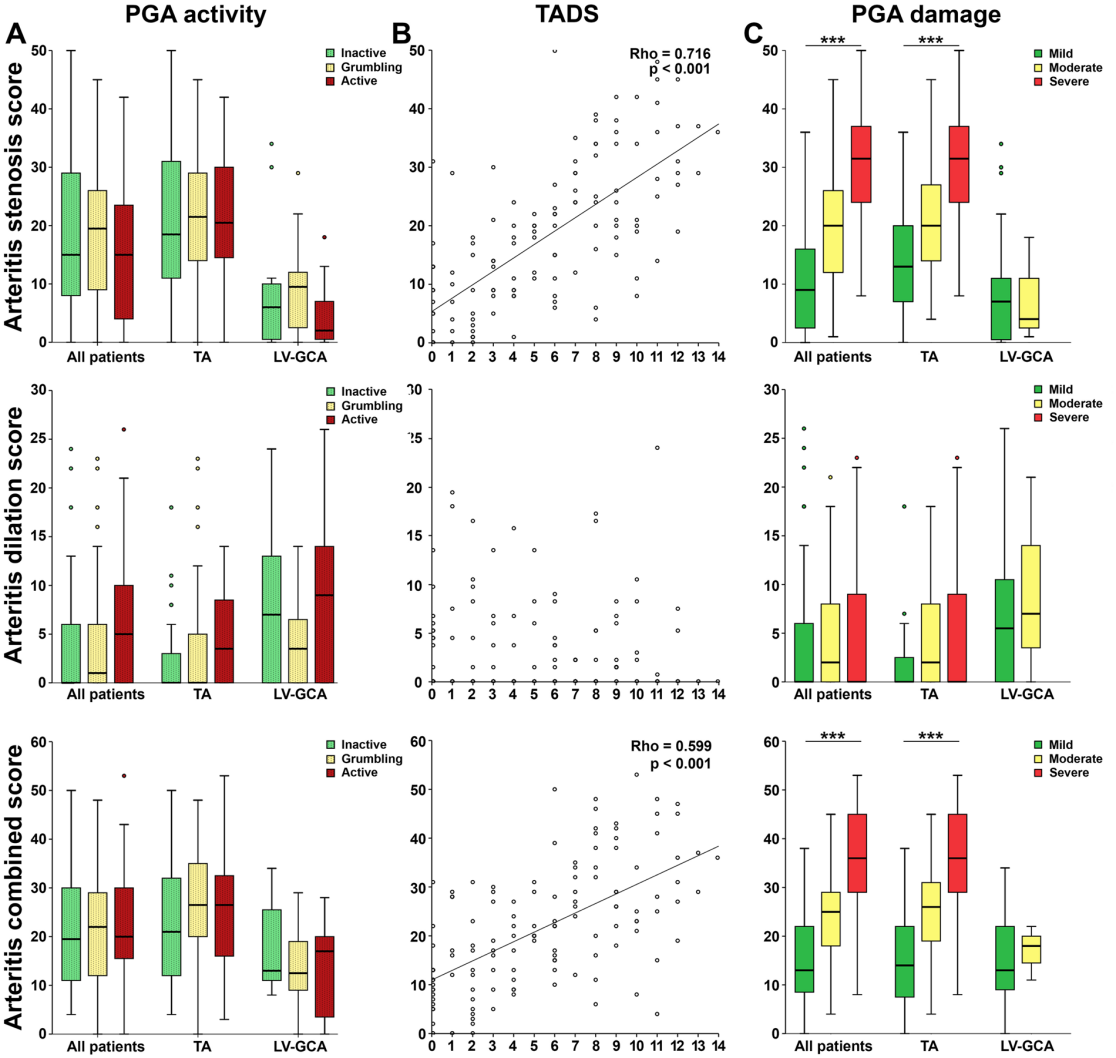
Cross-sectional validation of arterial injury scores against damage indices. (**A**) The stenotic, dilation and composite scores (y-axes) do not reflect disease activity, as assessed by physician global assessment (PGA) activity (x–axes). (**B**) The stenotic and composite scores correlate with damage as assessed by the Takayasu Arteritis Damage Score (TADS) (x-axes). (**C**) Stenotic and composite scores reflect damage as assessed by PGA damage (x-axes). Significance was evaluated by mixed-effect linear models (***p < 0.001).

To further assess the novel scoring algorithm, we analysed the relationship between the three scores and concurrent disease activity and damage. As expected, baseline scores did not correlate with CRP, ESR or any disease activity indices, either in TA or LV-GCA (Fig. 5A). Conversely, stenotic and composite scores in the whole cohort correlated with the Takayasu damage score (TADS) (rho = 0.716 and 0.627, respectively, p < 0.001), while the dilation score did not (Fig. 5B). Similar correlations between stenotic and composite scores and the TADS were found when the TA and LV-GCA groups were analysed separately (TA: rho = 0.655 and 0.587, p < 0.001, LV-GCA: 0.348, p = 0.041 and 0.407, p = 0.015). The stenotic and composite scores also reflected PGA damage in the whole cohort and in the TA subset (in all cases p < 0.001, Fig. 5C). In the LV-GCA subset only 4 patients exhibited moderate PGA damage and none had severe disease: accordingly, the correlation was not analysed. Overall, these data provide cross-sectional validation of the stenotic and composite scores against disease-related damage scores in the whole LVV cohort and in both the TA and LV-GCA subgroups.

### Longitudinal validation against activity measures

Sixty-seven patients underwent an additional 129 scans at our institute, and these were included in the longitudinal analysis (median interval 18 months, IQR 12–29). For longitudinal validation, changes in the novel scores between consecutive scans were calculated and related to matching disease activity measures using a mixed-effect linear model. The delta (Δ) of the stenotic, dilation and composite scores was scored as zero in 60, 115 and 53 scans, respectively. Of note, increased Δ stenotic, dilation and composite scores between scans were observed in patients with active disease as defined by ITAS2010, ITAS-CRP, ITAS-ESR and PGA (p < 0.005 for all indices), but not by NIH criteria (Table 2). This may reflect the more diverse elements of ITAS and the need to show changes in >2 of the 4 NIH criteria. Baseline scores were not related to any change in the scores during follow-up, either in the whole cohort (Table 2) or in the TA and LV-GCA subsets (not shown). These data provided an initial longitudinal validation of the novel scores, revealing rising scores in patients with active disease.

**Table 2:** Mixed effect linear model of the changes in ASS, ADS and ACS.

	Δ Stenosis score (ASS)		Δ Dilation scores (ADS)		Δ Composite score (ACS)	
	Difference from inactive pts	p-value	Difference from inactive pts	p- value	Difference from inactive pts	p-value
**NIH activity**	1.15 ± 0.81	0.162	0.34 ± 0.48	0.487	1.19 ± 1.04	0.257
**ITAS2010**	1.46 ± 0.50	**0.005**	0.35 ± 0.31	0.257	1.56 ± 0.66	**0.020**
**ITAS-CRP**	2.67 ± 0.72	**<0.001**	0.96 ± 0.39	**0.019**	3.27 ± 0.94	**<0.001**
**ITAS-ESR**	2.52 ± 0.78	**0.002**	1.12 ± 0.41	**0.008**	3.09 ± 1.02	**0.003**
**PGA activity**	p(ANOVA) = **0.004**		p(ANOVA) = **0.003**		p(ANOVA) = **0.002**	
Grumbling	0.47 ± 0.39	0.232	0.78 ± 0.23	**0.001**	1.26 ± 0.50	**0.001**
Active	2.48 ± 0.73	**0.001**	1.04 ± 0.46	**0.003**	2.84 ± 0.94	**0.004**
			**Regression slope**		**p-value**	
**ΔASS as a function of ASS**_**(t-1)**_^†^			−0.021 ± 0.018		0.276	
**ΔADS as a function of ADS**_**(t-1)**_^†^			0.056 ± 0.035		0.112	
**ΔACS as a function of ACS**_**(t-1)**_^†^			−0.004 ± 0.018		0.830	

LEGEND: CRP: C-reactive protein, ESR: Erythrocyte sedimentation rate, ITAS: Indian Takayasu Activity Score, NIH: National Institute of Health, PGA: physician global assessment.

^†^ASS_(t−1)_, ADS_(t−1)_, ACS_(t−1)_: ASS, ADS and ACS at the time −1, i.e. at the previous scan.

### Longitudinal validation against a reference radiological assessme**nt**

Further longitudinal validation of the new arterial scores established their accuracy against a reference assessment of disease evolution over time. In the absence of an accepted ‘gold standard’, we used a descriptive radiological assessment of arterial disease commonly applied in clinical practice. Each arterial stenosis or dilation was graded as “improved”, “stable” or “progressed” by two independent operators. Data from individual arteries were combined to obtain four distinct outcomes reflecting the evolution of stenotic and dilated lesions and the overall disease. The outcomes were separately defined as “improved”, if ≥1 artery improved and none progressed; “stable” if all arteries were unchanged, “mixed” if ≥1 artery improved and ≥1 progressed; “worsened” if ≥1 artery progressed and none improved (Supplementary Figs 2–4).

Interestingly, this detailed and standardised assessment showed that “reverse remodelling” is not rare in LVV. Stenotic disease improved in 33/129 scans, remained stable in 61/129, had a mixed evolution in 8/129 scans and worsened in 27/129 (Table 3). Therefore, the presence of arterial lesions and particularly stenosis, does not necessarily indicate irreversible damage. In contrast, arterial dilation never improved, remained stable in 111/129 and worsened in 17/129 scans. A single case showed mixed evolution of arterial dilation.

**Table 3:** Reference evolution of stenotic disease, dilation and overall disease.

	Stenotic Disease	Dilation	Overall Disease
**Improved**	33 (26%)	0 (0%)	30 (23%)
**Stable**	61 (47%)	111 (86%)	51 (40%)
**Mixed**	8 (6%)	1 (1%)	12 (9%)
**Worsened**	27 (21%)	17 (13%)	36 (28%)
**Disease progression**	35 (27%)	18 (14%)	48 (37%)

Improved scan: ≥1 lesions improved, 0 progressed. Stable scan: 0 lesions improved or progressed. Mixed scan: ≥1 lesions improved, ≥1 progressed. Worsened scan: 0 lesions improved, ≥1 progressed.

**Figure 6:**
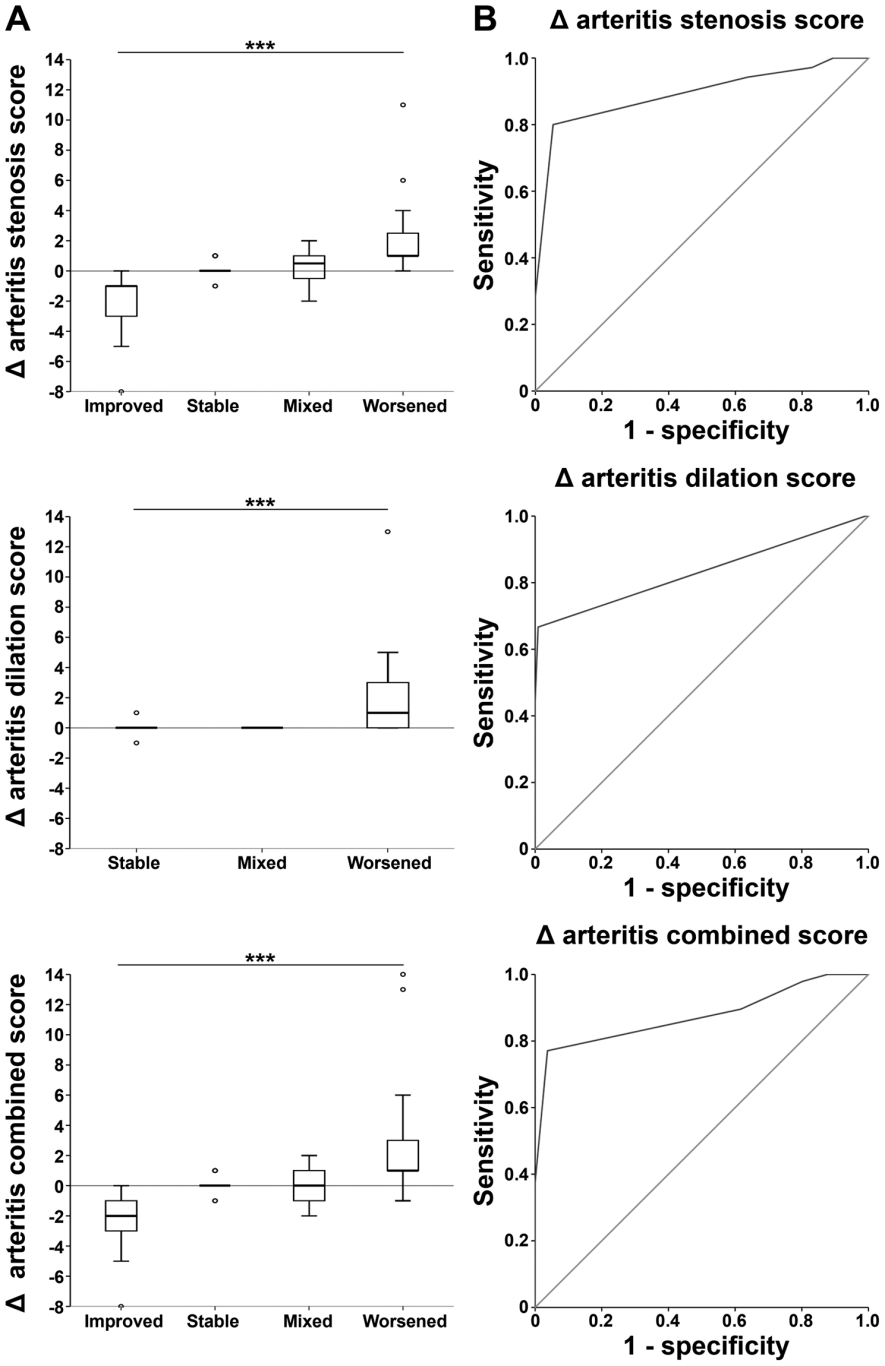
Longitudinal validation of the arterial injury scores and ROC analysis. (**A**) The change (Δ) in stenotic, dilation and composite scores (y-axes) reflects the outcomes of stenosis, dilation and overall disease respectively (x-axes). Significance was evaluated using mixed-effect linear models (***p < 0.001). (**B**) ROC curves of the Δ stenotic, dilation and composite scores for the detection of progressive arterial involvement.

Using mixed-effect linear models, we determined whether longitudinal changes in the novel stenosis, dilation and composite scores reflected the evolution of stenotic disease, dilation and overall disease identified as improved, stable, worsened or mixed in the qualitative analyses above. This analysis showed that changes in the three novel scores were closely related to disease outcomes (Supplementary Table 2 and Fig. 6A, p < 0.001 for all scores), demonstrating sensitive discrimination of improving, stable and worsening arterial disease.

### Receiver-operating characteristic (ROC) analyses

In order to identify changes in the novel scores that were predictive of arterial progression, the descriptive radiological assessment described above was used to stratify the follow-up imaging studies based on the presence of progression in at least one artery between consecutive scans (Table 3 and Supplementary Fig. 2). ROC analyses were then performed using the novel stenotic, dilation and composite scores, with the aim of distinguishing progressive and non-progressive disease (Fig. 6B).

Using a mixed-effect linear model, the area under the curve (AUC) of the change in the stenosis score was 0.949 (95%-IC: 0.883–0.995, p < 0.001). A change ≥1 had an observed sensitivity of 80% and specificity of 95% for detecting progressive stenosis. The AUC of the delta dilation score was 0.988 (95%-IC: 0.972–0.998, p < 0.001) and a Δ ≥ 1 gave a sensitivity of 67% and specificity of 99% for progressive dilation. Finally, the AUC for the change in composite score was 0.996 (95%-IC: 0.990–0.999, p < 0.001), with Δ ≥ 1 associated with overall progression (sensitivity 77%, specificity 96%). These data suggest that changes in all three scores have excellent specificity and good sensitivity for identifying arterial progression.

## Discussion

The development and validation of three new imaging-based scores is a first step towards a standardised, qualitative and quantitative definition of arterial involvement in LVV. The data presented demonstrate that the scores facilitate cross-sectional assessment of the pattern and extent of arterial injury and allow longitudinal monitoring of disease progression. In light of the importance put upon the development of validated scores for imaging assessment in LVV by EULAR and OMERACT^17,19^, we believe the new scores have significant potential, particularly for clinical trials, where they offer specific, relevant and quantitative primary outcome measures.

Although non-invasive imaging is essential for the management of LVV, debate continues concerning the optimal imaging modality for each stage. Likewise, it remains unclear whether confining imaging analysis to the arterial lumen is sufficient or whether extending it to include assessment of arterial wall inflammation and thickening is beneficial^17,23^. Although MRI studies of the arterial wall may reveal changes consistent with active arteritis including wall thickening and enhancement, the precise relationship with subsequent arterial remodelling is still unclear^24^. Recent studies suggest that combined FDG-PET/CT and MRA assessments may help to identify vasculitic lesions most likely to progress^25,26^ and confirmatory prospective studies are required.

In light of these uncertainties, and given the fact that luminal data derived from MRA or CTA studies directly reflect hemodynamic disturbance and the risk of end-organ ischemia, the new scores have so far been derived using exclusively luminal/angiographic data. This decision was made to allow the scores to: (a) be widely-applicable worldwide for multi-national clinical trials (MRA is much faster and less expensive than vessel wall imaging); (b) have conceptual similarity with the validated methods NASCET and ECST, and c) reflect haemodynamic alterations by scoring the degree of luminal stenosis or dilation. A similar approach has recently been followed by the American Heart Association to grade coronary artery dilation and aneurysms in Kawasaki disease^27^. Recognizing the potential importance of arterial wall data, an important additional strength of the proposed algorithm is its plasticity and capacity for future refinement. Thus, if luminal and wall analyses ultimately prove to be complementary, the current scoring algorithm can be adapted to incorporate arterial wall data for future studies.

In the current study, the three novel scores quantified arterial involvement and provided an immediate overview of both its severity and the pattern of arterial remodelling in the individual patient. Further, the analyses demonstrated that longitudinal change in each score specifically and sensitively identified improvement and deterioration in stenosis, dilation and overall arterial disease. Additionally, the scores allowed definition of the pattern and extent of arterial disease in groups of subjects. This was demonstrated by their ability to reflect significant differences between the TA and LV-GCA groups. Thus, application of the scores would improve identification of homogeneous groups for research studies including clinical trials. The stenosis and dilatation scores provide insight on the phenotype of remodelling, while the combined score reflects the total arterial damage burden. However, by combining the stenosis and dilatation scores there is a risk of one score ‘dampening’ the changes seen in the other and the potential impact of this requires further analysis in future studies. Likewise, dilatation and stenotic scores may be affected by other pathologies including diabetes mellitus, hypertension and atherosclerosis and this confounder must be born in mind when generating individual patient scores.

Longitudinal analysis revealed the dynamic nature of LVV lesions and highlighted the potential for reversibility. Consistent with previous reports^28,29^, improvement was almost uniquely observed in stenotic lesions. The ability to sensitively detect treatment-related changes in arterial stenosis is particularly important when considering application of the new scores in future clinical trials. Moreover, it suggests the scores represent more than just a damage index. However, established (≥6 months) changes in the scores might also be used to extend the TADS, which currently only records physical signs of arterial disease and hence under-estimates visceral artery involvement^30^.

Feasibility of use was an important objective in the development of the scores and we have established that they can be derived from CTA or MRA data. Imaging data acquisition requires a few minutes and is compatible with clinical schedules and technology available worldwide. The current data analysis time of 30–40 mins per scan is longer than a standard radiology assessment, suggesting the algorithm is best suited for clinical trials and research studies, rather than routine clinical assessments. However, the increasing availability of automated software for imaging analysis will reduce the analysis time.

Although comparable data was obtained by CTA and MRA, these modalities cannot yet be considered equivalent until inter-modality reproducibility is directly studied. Additional limitations include the use of a standardised descriptive radiological method for the evaluation of arterial disease evolution for the longitudinal validation of the novel scores. Although this approach has never been formally validated, it is the method most commonly used in clinical practice. To allow wider use of the novel scores, a limited core-set of 17 arteries most frequently involved in LVV were studied. Conceptually, this compares to the evaluation of a core-set of joints in rheumatoid arthritis. However, we accept that vasculitis might have involved arteries not included in the present study. A similar approach was adopted for the development of a combined arteritis damage score (CARDS), which used angiographic data to retrospectively investigate the CT or MRA studies of 41 TA and 55 GCA patients^31^. The CARDS quantifies arterial damage, reflecting stenosis, occlusion and dilatation in a single score. As also shown by the stenotic and composite scores herein, the median CARDS was significantly higher in GCA than TA^31^. The longitudinal phase of our current study and the use of three scores has now revealed correlation with disease activity and demonstrated that the new scores mirror disease evolution, reflecting both progressive arterial injury and lesion improvement.

In conclusion, a widely-applicable imaging-based scoring system which defines arterial involvement in LVV has been generated. Initial validation in a LVV cohort demonstrates the feasibility of the algorithm for a cross-sectional and longitudinal assessment of arterial disease. The new scoring system allows a standardised assessment of core features and facilitates the identification of homogeneous LVV subsets. We believe this is an important step towards fulfilling EULAR and OMERACT recommendations. We propose that the novel scores offer specific, objective and feasible arterial outcome measures which are essential for future clinical trials.

## Methods

The validated NASCET and ESCT methods for the assessment of carotid atherosclerosis were used as the basis for the development of the novel scores. They were specifically modified for LVV as follows:

1. A core-set of 17 arteries was defined on the basis of: (a) frequent involvement in LVV^10,22^ and (b) suitability for analysis by routine contrast-enhanced MRA and CTA (Table 1).
2. Each artery in the core-set received a stenosis and dilation score on the basis of both percentage and length of stenoses and dilations respectively (Fig. 1). Multiple lesions within the same artery were scored together, taking the worst percent stenosis/dilation and the sum of the lengths of stenotic lesions or dilations respectively.
3. Percent stenosis was calculated using a directly measured reference as required by NASCET whenever possible. Otherwise, a disease-free reference was estimated using ECST. The reference diameter was derived from a disease-free section of each artery (where specifically the arterial lumen did not appear to be affected by vasculitis), located either upstream or downstream to the plane of the most severe involvement and without significant branching between the two planes. Percent stenosis was graded using a five-point scale: 0 (no stenosis), 1 (<50% stenosis), 2 (50–70% stenosis), 3 (>70% stenosis) and 4 (occlusion/sub-occlusion).
4. Percent dilatation is calculated similarly to stenosis and then graded using a four-point scale, acknowledging that complete occlusion compromises downstream blood flow and threatens organ function more than an aneurysm. The reference for a dilated ascending aorta was estimated using age- and body surface area-adjusted normograms. Of note, to our knowledge, racially-adjusted normograms are not yet available.
5. Lesion length was graded using a four-point scale: 0 for focal lesions (length <10 mm), 1 for lesions above 10 and up to 30 mm, 2 for above 30 and up to 100 mm and 3 for >100 mm.
6. The sum of the stenosis or dilation scores of the 17 arteries represented the total Arteritis Stenosis Score and Arteritis Dilation Score. These were combined to generate the Arteritis Composite Score.
7. The scores were designed to exclude apparent improvement following vascular interventions. In the case of previous vascular intervention, the pre-interventional lesion length and percent stenosis or dilation were obtained. The score allocated was derived from the most severe arterial involvement in the pre- and post-interventional periods.
8. In the case of anatomical variants such as multiple renal arteries, the score assigned to the renal artery reflected the most severely affected. In the case of a common origin for two different arteries, (e.g., bovine branching of the brachiocephalic trunk or common origin of the coeliac and superior mesenteric arteries), the common tract was scored as if it belonged only to the largest artery (the brachiocephalic trunk or coeliac). In the event of an absent artery due to separate origins of its branches, the algorithm was adapted as follows: (i) absent brachiocephalic trunk, score omitted as the branches are included in the core-set, (ii) absent coeliac artery with separate origin of its branches from the aorta: the most severely affected artery was defined as the hypothetical coeliac. Table 1 summarises the final scoring algorithm.

### Study sample

The novel scoring system was applied to the LVV cohort followed at Hammersmith Hospital between 2010 and 2017. Supplementary Methods provide details of the enrolment criteria, the definitions used for TA and LV-GCA, imaging protocols and the multi-parametric clinical characterisation. Imaging was repeated whenever clinically indicated, using the same imaging modality (i.e. CTA or MRA) as the baseline study. To avoid circularity, imaging data made available for clinical characterisation did not include the novel score values. This research was approved by Imperial College Healthcare and performed in accordance with the Declaration of Helsinki. Informed written consent was obtained from all participants.

### Cross-sectional and longitudinal validation of the scoring system

Initial cross-sectional validation of the novel scores was performed using clinical measures of LVV damage (Takayasu Arteritis Damage Score (TADS) and Physician Global Assessment, (PGA) of damage). Longitudinal changes could not be validated with TADS or PGA damage, as they are insufficiently sensitive for assessing damage accrual. Therefore, longitudinal changes in the scores were validated against disease activity measures (NIH^11^, ITAS2010, ITAS-CRP, ITAS-ESR^13^, PGA activity and a 0–100 visual-analogue scale (VAS)), and against a typical radiological reference assessment of the evolution of stenosis, dilation and overall disease during follow-up. These three reference radiological assessments define disease evolution in four categories “improved”, “stable”, “mixed” and “worsened”, and the methodology is summarized in Fig. 2.

Two assessors compared each individual stenosis and dilation between consecutive scans, labelling lesions as “improved”, “stable” or “progressed”. Data were then combined to determine the global outcome of stenotic disease and dilation, respectively. Global outcome was labelled as “improved”, if ≥1 artery improved and none progressed; “stable” if all arteries were unchanged, “mixed” if ≥1 artery improved and ≥1 progressed; “worsened” if ≥1 artery progressed and none improved. The “mixed” category accounts for simultaneous improvement and worsening of lesions within the same patient. The outcome of overall disease was defined by including both stenoses and dilations. To ensure independence between the two different evaluations, longitudinal assessment of the stenosis, dilation and composite scores and the derivation of the three reference assessments were performed at different times. A receiver-operating characteristic (ROC) analysis was subsequently performed to assess the accuracy of measured changes in the stenotic, dilation and composite scores for the evaluation of disease progression.

### Statistical analysis

Non-parametric analysis was performed: quantitative variables are presented as median and inter-quartile ranges (IQR). Mann-Whitney and Kruskal-Wallis tests were used to compare scalar variables at baseline. Spearman rank correlation coefficients were calculated for the correlation analyses. Relationships between categorical variables were investigated by the χ^2^ test or the Fisher’s Exact Test, as appropriate. Intra- and inter-observer reliability were calculated using the intra-class correlation coefficient^32^ based on a 2-way random effect model in a subset of 18 and 23 scans, randomly selected from our cohort. Previously established categories for expressing levels of reliability were used^32^. For longitudinal analysis of the changes in the scores, a mixed-effect linear model analysis was performed, to account for variable numbers of scans between patients. Similarly, the area under the ROC curve was calculated using a confidence interval of 95% (95%-IC) using a mixed effect linear model. A two-tailed p-value ≤0.05 was considered statistically significant. Statistical analysis was performed with IBM® SPSS® statistic, version 20 and R statistics.

## Electronic supplementary material

Supplementary material
