# Supplementary material for: Novel Angiographic Scores for evaluation of Large Vessel Vasculitis
Source: Sci Rep. 2018 Oct 29;8:15979. doi: 10.1038/s41598-018-34395-7 (PMC6206009; doi:10.1038/s41598-018-34395-7)
Supplement: Supplementary file 1 — Supplementary material [file 41598_2018_34395_MOESM1_ESM.docx]

**Novel Angiographic Scores for evaluation of Large Vessel Vasculitis**

Enrico Tombetti, Claudia Godi, Alessandro Ambrosi, Frances Doyle, Alana Jacobs, Allan Kiprianos, Taryn Youngstein, Katie Bechman, Angelo A Manfredi, Ben Ariff and Justin C Mason.

**Supplementary material**

**SUPPLEMENTARY METHODS**

**Enrolment criteria and patient definition**

The study cohort is composed of patients with TA or LV-GCA followed at Hammersmith Hospital (Imperial College Healthcare NHS Trust) between 2010 and 2017. TA patients were defined by either: a) the American College of Rheumatology (ACR) criteria^1^, or b) age of disease onset ≤50 years and typical TA findings at CTA, MRA or positron-emission tomography (PET). LV-GCA patients were >50 years at onset with imaging evidence of LVV. All patients were extensively evaluated to exclude LVV mimics or potential differential diagnoses. LVV patients with CTA or MRA scans suitable for inclusion in the analysis were enrolled: scans were required to have volumetric acquisition and to encompass the carotid, subclavian-axillary axes and common iliac arteries. In 8 of the 139 LVV patients identified scan quality did not meet enrolment criteria. The final sample comprised 131 patients (96 TA, 35 LV-GCA) (**Supplementary Table 1**).

Patients were enrolled at the time of imaging assessment and then followed prospectively: imaging was repeated whenever clinically indicated, using the same imaging modality (i.e. CTA or MRA) as the baseline study. Median interval between consecutive scans: 18 months, IQR 12-29. These follow-up imaging studies were included in the longitudinal validation of the scoring algorithm.

**Imaging protocols**

MRA was performed using a 1.5T MR whole-body scanner (Achieva, Philips, The Netherlands) with phased-array coils (Philips XL SENSE torso coil, 16 channels). First-pass MRA dynamic sequences targeted on the aorta (Fast-Field Echo coronal 3D T1-weighted images; FOV FHxRLxAP = 520x485x96mm; Acq Matrix = 472x445; Recon Matrix = 560; Acq Voxel = 1.10/1.09/1.60mm; Rec Voxel = 0.94mm; Slice thickness = 1.6mm; TR/TE = 4/1.37; Acquisition time = 27.5s) were performed on supine patients during contrast media infusion (Gadovist® 1nmol/ml Bayer, Germany; dose: 0.1ml/Kg) injected at 1.5ml/s, followed by 25ml of saline at 1.5ml/s. Delay time was customised by “bolustrak”. The acquisition was positioned coronally. In the case that multiple MRA scans were required to cover the different vascular districts due to particularly large body size, data were derived from multiple scans performed within 2 weeks of each other.

CTA was performed with a Siemens Somatom Definition AS+ MDCT scanner (Siemens Healthcare, Erlagen, Germany) with a 128 x 0.6 mm detector collimation. The following parameters were used: reference tube voltage 120 kV (100-140 kV was used, selected with CAREkV depending on body mass), reference tube current-time product 120 mAs (varying depending on body size using CAREDose), pitch 0.6 and utilizing iterative reconstruction (SAFIRE 3). Reconstructed slice width 1mm. After 10 ml of i.v. saline, contrast-medium (Omnipaque 300, GE Healthcare, UK; minimum 80 ml) was infused, followed by 40 ml of saline at 3 ml/h. Delay time was customized by “bolustrak”. All imaging studies were jointly evaluated by two observers.

**Clinical assessment**

A thorough clinical assessment was performed within two months of the baseline and follow-up imaging studies (**Supplementary Table 1**). Disease activity was evaluated using the NIH criteria^2^, the ITAS-2010, ITAS-C-reactive protein (CRP) and ITAS-erythrocyte sedimentation rate (ESR)^3^. Given the relative limitations of NIH criteria and ITAS, disease activity was also evaluated by Physician Global Assessment (PGA) of activity (“inactive”, “grumbling/persistent”, “active”^3^) and a visual-analogue 0-100 scale (VAS). LVV damage was evaluated by PGA damage (“mild”, “moderate”, “severe”) and by the Takayasu Arteritis Damage Score (TADS)^4^. CGA cephalic involvement was defined by the presence of any of the following features: new-onset headache (not otherwise explained), scalp tenderness, jaw claudication, ischemic visual episodes, temporal artery tenderness to palpation or decreased pulsation unrelated to proximal arteriopathy, positive temporal artery biopsy or temporal artery ultrasonography positive for halo sign.

Two rheumatologists jointly evaluated disease activity and damage. In the absence of specific indices for LV-GCA, those developed for TA were applied. PGA of activity and damage for LV-GCA patients was performed, taking into account specific features of GCA, including polymyalgia rheumatica, headache not otherwise explained, jaw claudication, scalp tenderness and recent episodes of amaurosis fugax or optic neuritis for disease activity and optic neuritis alone for damage. State-of-the art follow-up of LVV frequently requires imaging: these data, which did not include the novel scores, were made available to the physician at the time of clinical assessment.

**Statistical analysis**

Non-parametric analysis was performed: quantitative variables are presented as median and inter-quartile ranges (IQR). Mann-Whitney and Kruskal-Wallis tests were used to compare scalar variables at baseline. Spearman rank correlation coefficients were calculated for the correlation analyses. Relationships between categorical variables were investigated by the χ^2^ test or the Fisher’s Exact Test, as appropriate. Intra- and inter-observer reliability were calculated using the intra-class correlation coefficient^5^ based on a 2-way random effect model in a subset of 18 and 23 scans, randomly selected from our cohort. Previously established categories for expressing levels of reliability were used^5^. For longitudinal analysis of the changes in the scores, a mixed-effect linear model analysis was performed, to account for variable numbers of scans between patients. Similarly, the area under the ROC curve was calculated using a confidence interval of 95% (95%-IC) using a mixed effect linear model. A two-tailed p-value ≤0.05 was considered statistically significant. Statistical analysis was performed with IBM® SPSS® statistic, version 20 and R statistics.

**REFERENCES**

1. Arend WP, Michel BA, Bloch DA, Hunder GG, Calabrese LH, Edworthy SM, Fauci AS, Leavitt RY, Lie JT, Lightfoot Jr. RW, et al. The American College of Rheumatology 1990 criteria for the classification of Takayasu arteritis. *Arthritis Rheum*. 1990;33:1129–1134.

2. Kerr GS, Hallahan CW, Giordano J, Leavitt RY, Fauci AS, Rottem M, Hoffman GS. Takayasu arteritis. *Ann Intern Med*. 1994;120:919–929.

3. Misra R, Danda D, Rajappa SM, Ghosh A, Gupta R, Mahendranath KM, Jeyaseelan L, Lawrence A, Bacon PA, Indian Rheumatology Vasculitis group. Development and initial validation of the Indian Takayasu Clinical Activity Score (ITAS2010). *Rheumatol*. 2013;52:1795–1801.

4. Rajappa SM. Outcome of vascular interventions in Takayasu Arteritis using the Takayasu Arteritis Damage Score. *Arthritis Rheum*. 2011;63:150.

**SUPPLEMENTARY FIGURES**

**
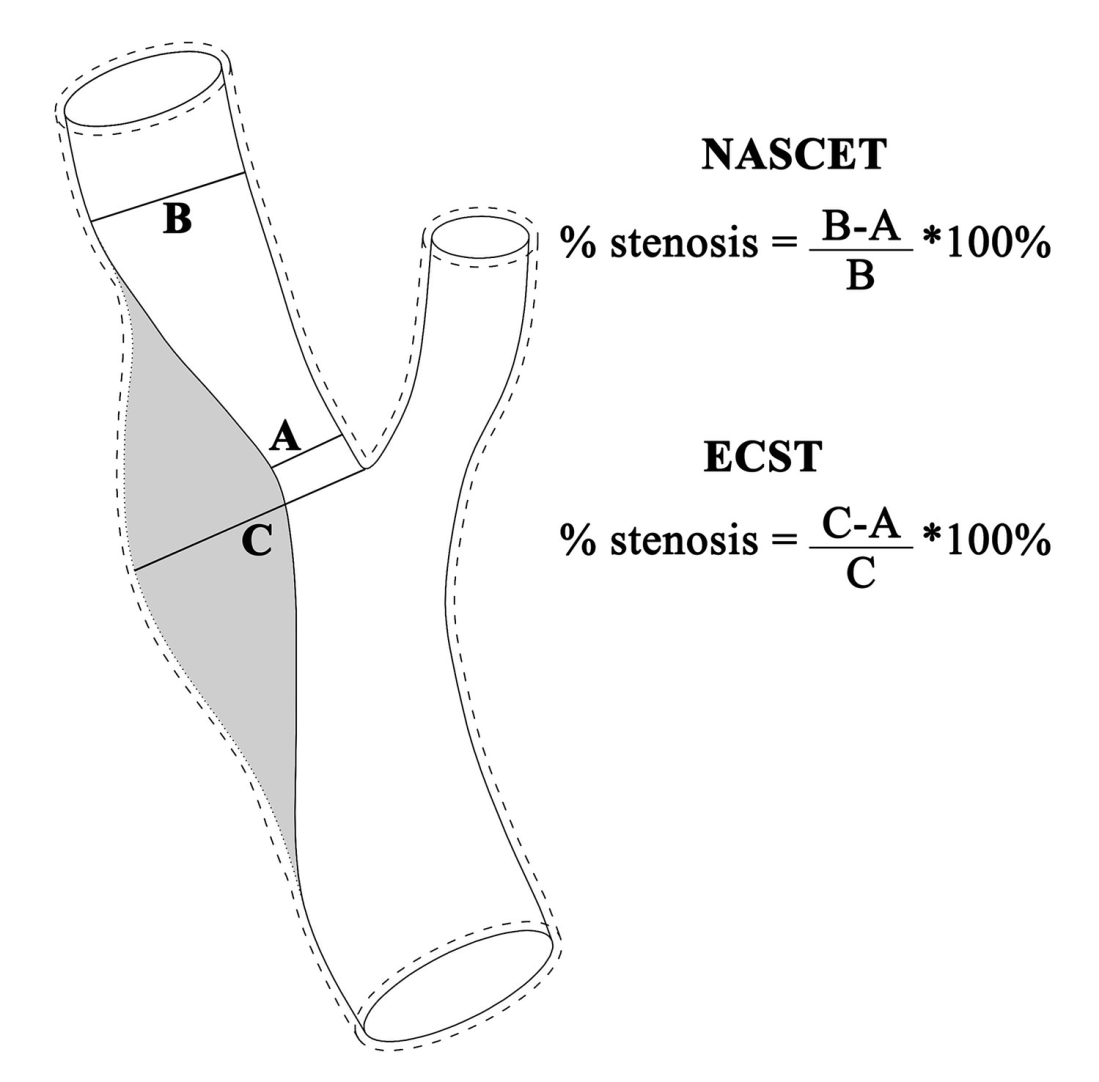
**

**Supplementary Figure 1. The NASCET and ECST methods.**

The NASCET and ECST methods are validated for the assessment of carotid artery atherosclerosis severity. The NASCET method uses as a reference a direct measure of the luminal diameter in a plane not involved by disease, while the ECST utilizes the estimated normal luminal diameter.

**
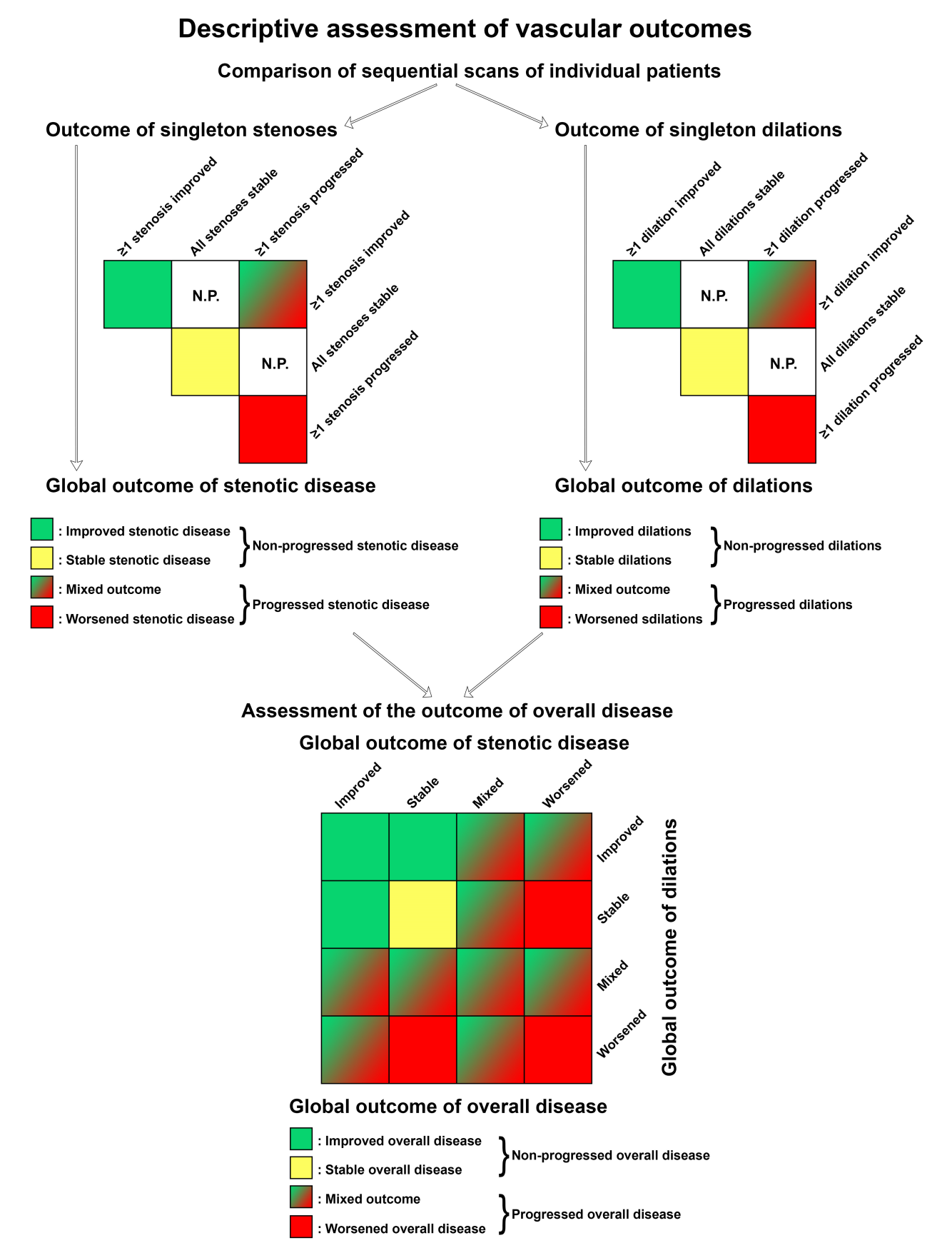
**

**Supplementary Figure 2. Descriptive assessment of vascular outcomes.**

The descriptive assessment of vascular outcomes represented the reference for the longitudinal validation of the scores. Outcomes of each stenosis or dilation were classified as “improved”, “stable” or “progressed”. By combining the outcomes of individual lesions in each patient, the global outcomes of stenotic disease, dilation and overall disease were derived. Progression of stenotic disease, dilation and overall disease was defined as the presence of ≥1 lesion undergoing progression, as shown. (N.P: not possible).

**
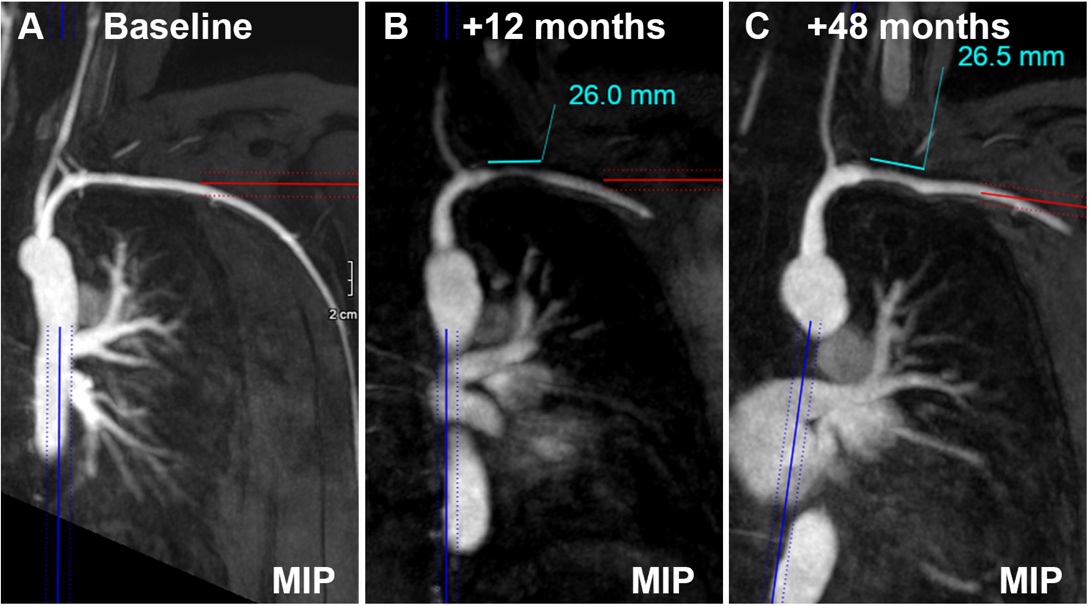
**

**Supplementary Figure 3. Detection of disease progression and improvement.**

**A**: No obvious stenosis in the left subclavian artery was observed at baseline. **B**: after 12 months, a new stenosis was revealed. Tocilizumab therapy was initiated. **C**: reverse remodeling with improvement of the stenosis was seen 48 months after baseline.

**

**

**Supplementary Figure 4.** **New appearance of aortic dilation.**

**A**: Overview of the ascending aorta at baseline maximum intensity projection (MIP) images. **B**: measure of the largest diameter of the ascending aorta at baseline. **C**: multi-vessel arterial dilation was observed after 19 months, particularly in the ascending aorta (MIP images). **D**: after 19 months, the largest diameter of the aorta was significantly wider than at baseline.

**Supplementary Table 1: Patient demographics**

| ***Qualitative variables*** | **All patients**  **(N=131)** | **TA**  **(N=96)** | **LV-GCA**  **(N=35)** | **p-value** | |
| --- | --- | --- | --- | --- | --- |
| Gender (F:M) | 123:8 | 91:5 | 32:3 | 0.440 | |
| Scan type (MRA:CTA) | 114:17 | 85:11 | 29:6 | 0.390 | |
| Class of vascular involvement:  I  IIa  IIb  III  IV  V | 11 (8%)  4 (3%)  12 (9%)  15 (11%)  3 (3%)  85 (65%) | 6 (6%)  3 (3%)  10 (10%)  11 (11%)  2 (2%)  64 (67%) | 5 (14%)  1 (3%)  2 (6%)  4 (11%)  2 (6%)  21 (60%) | 0.567 | |
| Steroids | 82 (63%) | 54 (57%) | 28 (80%) | **0.015** | |
| Immunosuppressive agents  Azathioprine  Methotrexate  Mycophenolate mofetil  Cyclophosphamide | 69 (55%)  10 (8%)  52 (40%)  7 (5%)  1 (1%) | 51 (53%)  9 (9%)  38 (40%)  5 (5%)  1 (1%) | 18 (51%)  1 (3%)  14 (40%)  2 (6%)  0 | 0.863  0.214  0.966  0.909  0.544 | |
| Biologic agents  TNFα-blockers  Tocilizumab | 7 (6%)  6 (5%)  1 (1%) | 7 (7%)  7 (7%)  1 (1%) | 0 | 0.189 | |
| Active disease  NIH criteria  ITAS2010  ITAS-CRP  ITAS-ESR | 22 (17%)  34 (26%)  20 (15%)  22 (17%) | 13 (14%)  23 (24%)  11 (11%)  12 (12%) | 9 (26%)  11 (31%)  9 (26%)  10 (28%) | 0.105  0.425  **0.048**  **0.042** | |
| PGA activity  Inactive  Grumbling/persistent  Active | 58 (44%)  42 (32%)  31 (24%) | 46 (48%)  30 (31%)  20 (21%) | 12 (34%)  12 (34%)  11 (31%) | 0.306 | |
| PGA damage  Mild  Moderate  Severe | 68 (52%)  37 (28%)  26 (20%) | 36 (37%)  34 (35%)  26 (27%) | 32 (91%)  3 (9%)  0 | **<0.001** | |
| ***Scalar variables (median and IQR)*** |  |  |  |  | |
| Age (years) | 51 (33-63) | 41 (30-54) | 64 (58-70) | **<0.001** | |
| Disease duration (years) | 4 (1-9) | 5 (2-12) | 2 (1-6) | **<0.001** | |
| Scan-to-clinical evaluation (days) | 27 (2-53) | 27 (1-54) | 27 (3-42) | 0.948 | |
| Steroid dose (mg/day) | 5.0 (0-10.0) | 5.0 (0-10.0) | 10 (2-30) | **0.002** | |
| ESR (mm/h) | 25 (13-47) | 25 (13-47) | 23 (10-44) | 0.577 | |
| Serum C-reactive protein (mg/L) | 5.0 (2.0-14.3) | 5.0 (2.0-13.6) | 6.0 (1.9-25.4) | 0.436 | |
| VAS activity (0-100) | 15 (5-31) | 15 (5-28) | 20 (7-40) | 0.270 | |
| TADS | 5 (2-9) | 7 (4-9) | 1 (0-4) | **<0.001** | |
| Arteritis Stenosis Score | 16 (8-27)  range: 0-50 | 16 (8-27)  range: 0-50 | 7 (1-11)  range: 0-34 | **<0.001** | |
| Arteritis Dilation Score | 0 (0-7)  range: 0-26 | 0 (0-7)  range: 0-26 | 6 (0-11)  range: 0-26 | **0.013** | |
| Arteritis Composite Score | 21 (12-29)  range: 0-53 | 21 (12-29)  range: 0-53 | 13 (9-22)  range: 0-34 | **<0.001** | |
| LEGEND: ESR: Erythrocyte sedimentation rate, ITAS: Indian Takayasu Activity Score, NIH: National Institute of Health, TADS: Takayasu Arteritis Damage Score, VAS: visual-analogue scale. The p values reflect the statistical analysis of TA compared to LV-GCA. | | | | |  |

**Supplementary Table 2: Changes in ASS, ADS and ACS reflect the reference evolution of stenosis, dilation and overall disease** (N=129 scans, 67 patients).

|  | **ΔASS** | |
| --- | --- | --- |
|  | **Difference from “improved” cases** | **p-value** |
| **Evolution of stenotic disease*** | p (ANOVA) **<0.001** | |
| **Stable**  **Mixed**  **Worsened** | 2.14 ± 0.30  2.34 ± 0.24  4.02 ± 0.36 | **<0.001**  **<0.001**  **<0.001** |
|  | **ΔADS** | |
|  | **Difference from “stable” cases** | **p-value** |
| **Evolution of dilation†** | p (ANOVA) **<0.001** | |
| **Mixed**  **Worsened** | 0.31 ± 0.58  1.97 ± 0.19 | 0.598  **<0.001** |
|  | **ΔACS** | |
|  | **Difference from “improved” cases** | **p-value** |
| **Evolution of overall disease*** | p (ANOVA) **<0.001** | |
| **Stable**  **Mixed**  **Worsened** | 2.22 ± 0.43  2.36 ± 0.66  4.64 ± 0.47 | **<0.001**  **<0.001**  **<0.001** |
| *: mean differences ± standard deviation from cases with improved evolution; †: mean differences ± standard deviation from cases with stable evolution | | |
